# Effects of in Ovo Feeding With Glycosaminoglycans and Vitamins on Intestinal Morphometry and Cadherin‐1 Gene Copy Abundance in Broilers

**DOI:** 10.1111/asj.70186

**Published:** 2026-05-01

**Authors:** Elaine Talita Santos, Silvana Martinez Baraldi‐Artoni, Jean Kaique Valentim, Rodrigo Garófallo Garcia, Peter Ferket, Ramon Diniz Malheiros, Melody Martins Cavalcante Pereira, Sarah Sgavioli

**Affiliations:** ^1^ Postgraduate Program in Animal Science São Paulo State University Jaboticabal São Paulo Brazil; ^2^ Department of Animal Production, Institute of Animal Science Federal Rural University of Rio de Janeiro Seropédica Rio de Janeiro Brazil; ^3^ Faculty of Agricultural Sciences Universidade Federal da Grande Dourados Dourados Mato Grosso do Sul Brazil; ^4^ Department of Poultry Science North Carolina State University Raleigh North Carolina USA; ^5^ Graduate Program in Animal Science Pontifícia Universidade Católica do Paraná Curitiba Paraná Brazil

**Keywords:** cadherin‐1, glycosaminoglycans, in ovo feeding, intestinal morphometry

## Abstract

This study evaluated the effects of in ovo feeding with glycosaminoglycans and vitamins, combined with amphotericin and gentamicin, on incubation traits, intestinal morphometry, organ development, and cadherin‐1 gene relative abundance in broilers. Fertile Ross 308 breeder eggs were randomly assigned to the following five treatments: uninjected eggs; eggs injected with a solution containing amphotericin, gentamicin, ascorbic acid, and pyridoxine; the same solution supplemented with chondroitin sulfate; glucosamine sulfate; or both glycosaminoglycans. Hatching rate, embryo mortality, and total mortality were not affected by treatments (*p* > 0.05). Broilers from the solution‐based treatment showed higher relative heart weight at 1 day of age (*p* < 0.05). Intestinal morphometric parameters increased with age in both the jejunum and ileum. In ovo feeding modulated villus height, crypt depth, and villus height–crypt depth ratio, particularly in the ileum during early posthatch development. The relative abundance of the cadherin‐1 (CDH1) gene was influenced by the interaction between in ovo feeding and age, with higher values observed in broilers receiving glycosaminoglycan‐supplemented solutions at specific posthatch periods. In conclusion, in ovo feeding with glycosaminoglycans and vitamins modulated early intestinal development and relative heart weight without impairing hatchability in broilers.

## 1 Introduction

Glycosaminoglycans, including chondroitin and glucosamine, are bioactive compounds widely recognized for their anti‐inflammatory properties, which contribute to reducing joint deterioration and preventing bone injury (Sgavioli et al. 2017). Among these compounds, glucosamine sulfate stands out as a precursor of chondroitin sulfate and a key substrate for glycosaminoglycan synthesis (Chang et al. 2006). This dual role enables glucosamine sulfate to stimulate glycosaminoglycan production while reducing their degradation, thereby providing substantial benefits for skeletal health (Martins et al. 2023).

Several studies have demonstrated that glucosamine sulfate supplementation alleviates leg disorders (Sgavioli et al. 2017) and supports the structural development of bones and joints in broilers, highlighting its potential to improve poultry performance (Santos et al. 2019; Martins, Santos Neto, Gomides, et al. 2020; Martins et al. 2023). These findings reinforce the relevance of glycosaminoglycans as functional compounds capable of modulating skeletal integrity in fast‐growing poultry genotypes.

Beyond their effects on skeletal tissues, cadherins are single‐chain transmembrane glycoproteins that mediate calcium‐dependent cell–cell adhesion and play a central role in tissue development (Yagi and Takeichi 2000). Cadherins are involved in key biological processes such as cell proliferation, differentiation, and cell survival (Larue et al. 1996; Gumbiner 2005). These mechanisms are essential for maintaining tissue integrity and are also associated with both skeletal and intestinal development, underscoring the importance of understanding how nutritional strategies, such as in ovo feeding, may modulate cadherin‐related pathways.

In addition, the anti‐inflammatory effects of chondroitin and glucosamine sulfates extend beyond skeletal health to the intestinal environment. Experimental studies have shown that these compounds enhance epithelial defenses (Castrogiovanni et al. 2016), improve intestinal histomorphometry, promote mucin biosynthesis, and modulate the intestinal microbiota (Segarra et al. 2016; Salvatore et al. 2000; Martins, dos Santos Neto, Noleto‐Mendonça, et al. 2020; Liu et al. 2017). Collectively, these findings suggest that glycosaminoglycans may exert both systemic and localized effects, supporting intestinal structure and function in broilers.

The nutritional composition of eggs plays a critical role in embryonic development and early posthatch performance (de Paula et al. 2024). In this context, in ovo feeding has emerged as a promising strategy to enhance embryonic development and increase posthatch nutrient availability (Quadros et al. 2021; Sgavioli et al. 2015; Sgavioli et al. 2016; Sgavioli et al. 2022). This technique involves the injection of tailored nutrient solutions, commonly composed of vitamins, minerals, amino acids, probiotics, or their combinations, directly into eggs during incubation (Yair and Uni 2011; Roto et al. 2016; Sgavioli et al. 2019).

Previous studies have demonstrated that in ovo feeding with glycosaminoglycans and vitamin C improves bone development and reduces mortality during incubation (Santos et al. 2018), while pyridoxine administration in ovo increases the relative weight of hatchlings (El‐Kholy et al. 2019). However, although the effects of glycosaminoglycans and ascorbic acid on bone characteristics (Santos et al. 2018) and the influence of dietary glycosaminoglycans on intestinal histomorphometry in broilers (Martins, dos Santos Neto, Noleto‐Mendonça, et al. 2020) have been previously investigated, limited information is available regarding the combined effects of glycosaminoglycans, vitamins, antibiotics, and antifungals administered via in ovo feeding.

Such combinations may represent a novel strategy to support both intestinal and organ development during early posthatch life, while modulating the expression of cadherin‐1, a gene that plays a critical role in tissue formation and maintenance (Yagi and Takeichi 2000).

Therefore, this study hypothesized that in ovo feeding with a combination of glycosaminoglycans, ascorbic acid, pyridoxine, amphotericin, and gentamicin can influence intestinal and organ development in broilers, increase cadherin‐1 gene relative abundance, and achieve these effects without compromising hatchability or embryo survival. The findings of this study aim to contribute to the advancement of in ovo feeding strategies as tools to optimize early development and performance in commercial broiler production.

## 2 Materials and Methods

### 2.1 Ethics Statement and Incubation Conditions

This study was approved by the Local Committee of Ethics in Animal Use (case no. 011424/13). A total of 640 fertile eggs from 47‐week‐old Ross 308 breeders were incubated in four Chore Time CTB incubators (Milford, IN), each with a capacity of 180 eggs. The incubators were equipped with automatic temperature, humidity, and egg‐turning control systems. Incubation conditions were set at an initial temperature of 37.5°C and relative humidity of 60%, which were gradually adjusted to 36.5°C and 70%, respectively, by the end of the 21‐day incubation period.

### 2.2 Experimental Design and in Ovo Treatments

At 17.5 days of incubation, eggs were individually weighed and randomly distributed into five treatment groups (128 eggs per treatment), ensuring uniform egg weight among treatments (average egg weight: 67 ± 1.16 g). Each treatment was equally represented in all incubators, with 32 eggs per incubator.

In ovo feeding was performed by injecting 0.6 mL of the assigned solutions into the amniotic fluid using a 21‐gauge needle, as identified by candling (Tako et al. 2004). The treatments were as follows:

1. Control (uninjected): eggs without perforation or injection.
2. Solution‐based (SB): amphotericin (500 μL), gentamicin (2 μL), ascorbic acid (1 g), and pyridoxine (10 mg).
3. SB + chondroitin: SB solution supplemented with 50 mg of chondroitin sulfate.
4. SB + glucosamine: SB solution supplemented with 100 mg of glucosamine sulfate.
5. SB + chondroitin + glucosamine: SB solution supplemented with 50 mg of chondroitin sulfate and 100 mg of glucosamine sulfate.

Chondroitin sulfate (C₁₄H_2_₁NO₁₄S)_n_ (91.35% purity; Infiniti Nutraceuticals Inc.) and potassium glucosamine sulfate [(C₆H₁₄NO₅)_2_SO₄ × 2KCl; 15.7% sulfate content; Zhejiang Freemen Inc.] were used as glycosaminoglycan sources.

### 2.3 Posthatch Management

After hatching, 320 male chicks (64 per treatment) with body weights close to the experimental unit mean were selected and housed in battery cages. Birds were distributed into 40 pens (8 birds per pen), totaling eight replicates per treatment, and reared for 14 days. Environmental temperature and relative humidity were automatically controlled according to the Ross Broiler Management Guide (Ross 2014), with temperatures ranging from 27°C to 34.1°C and relative humidity from 15% to 73.36%. Feed and water were provided ad libitum. Diets were formulated according to Rostagno et al. (2011) to meet nutritional requirements, providing 2800 kcal kg^−1^ metabolizable energy, 21.27% crude protein, and adequate levels of methionine, lysine, calcium, and phosphorus.

### 2.4 Hatching Performance and Mortality

Hatching rate was calculated as the proportion of hatched chicks relative to the number of incubated eggs. Embryo mortality was classified according to developmental stage as early (1–7 days), intermediate (8–14 days), or late (15–21 days) incubation.

### 2.5 Organ Weights

At 1, 7, and 14 days posthatch, six birds per treatment (90 birds total) with body weights close to the treatment mean were selected. Birds were fasted for 8 h, weighed, and euthanized by CO_2_ stunning followed by exsanguination. The heart, liver, and bursa of Fabricius were excised and weighed using an analytical balance (Toledo, Adventurer AR1530, São Bernardo do Campo, SP, Brazil). Relative organ weights were expressed as percentages of body weight.

### 2.6 Intestinal Morphometry

Segments (0.5 cm) from the mid‐jejunum and mid‐ileum were collected, longitudinally opened, rinsed with NaCl solution (9 g L^−1^), and fixed in buffered formalin (90 mL L^−1^). Samples were subsequently stored in 70% ethanol at 4°C. Tissue sections were prepared using a cryostat at −20°C (CM30505, Leica Microsystems, Wetzlar, Germany), cut into 10‐μm sections, and stained with hematoxylin and eosin.

Morphometric evaluations included villus height, villus tip width, villus base width, crypt depth, muscularis thickness, and villus height‐to‐crypt depth ratio. Measurements were obtained using an optical microscope (Eclipse E600, Nikon Corp.) coupled to a digital camera (XC77E, Sony Corp.) and analyzed using AmScope image analysis software. Ten measurements were taken per image from five images per section.

### 2.7 Cadherin‐1 Gene Copy Abundance

The left tibia from each bird was collected, homogenized in phosphate‐buffered saline, and centrifuged to remove debris. Genomic DNA was extracted using the Gentra Puregene Cell Kit following the manufacturer's instructions.

The relative abundance of the cadherin‐1 (CDH1) gene was determined by quantitative PCR using specific primers (forward: TGAGAAGCAGATACTGAGCATTGTG; reverse: GCTCATCTTGGCCCCTTATCTC). Results were expressed as relative gene copy abundance and log_10_‐transformed prior to statistical analysis.

### 2.8 Statistical Analysis

Data were analyzed using SAS software (SAS Institute Inc. 2012). Residuals were evaluated for normality using the Shapiro–Wilk test, and outliers were assessed prior to analysis. Data were analyzed using the PROC MIXED procedure, with age considered a repeated measure. The statistical model included the fixed effects of in ovo feeding (IOF), age (AG), and their interaction:

Y=μ+IOF+AG+IOF×AG+ε

where Y represents the dependent variable, μ is the overall mean, IOF is the effect of in ovo feeding, AG is the effect of age, and ε is the random error term. The covariance structure was selected based on the lowest corrected Akaike information criterion (AICC) value (Wang and Goonewardene 2004). Mean comparisons were performed using Tukey's test. Hatching rate, embryo mortality, and total mortality were analyzed using the chi‐square test. Statistical significance was declared at *p* < 0.05.

## 3 Results

No significant effects of the treatments during incubation were observed on hatching rate, embryo mortality, or total mortality (*p* > 0.05; Table 1). There was a significant interaction between in ovo feeding and age on relative heart weight (*p* = 0.0465). Broilers from the solution‐based treatment group presented greater relative heart weight at 1 day of age compared with the other treatments.

**TABLE 1:** Effects of in ovo feeding on hatchability, embryonic mortality at different incubation stages, and total embryonic mortality in broiler chickens.

In ovo feeding (IOF)	Hatching	Embryo mortality (%)			Total mortality (%)
	(%)	1–7	7–14 days	14–21	
Uninjected egg	72.73	0.00	2.27	13.64	27.27
Solution based (SB)	74.29	2.86	0.00	20.00	25.71
SB/chondroitin	71.43	0.00	5.71	17.14	28.57
SB/glucosamine	60.00	0.00	0.00	28.57	40.00
SB/chondroitin/glucosamine	68.57	2.86	5.71	14.29	31.43
Probability chi‐square	0.2202	0.2873	0.2650	0.2202	0.2202

*Note:* No significant differences were observed according to the chi‐square test at the 5% probability level. Total mortality represents cumulative embryonic mortality during incubation.

Body weight, as well as the relative weights of the liver and bursa of Fabricius, was significantly affected by age (*p* < 0.0001), with values increasing as birds aged, regardless of in ovo feeding treatment (Table 2).

**TABLE 2:** Effects of in ovo feeding on body weight and relative weights of the heart, liver, and bursa of Fabricius in broiler chickens at 1, 7, and 14 days of age.

	In ovo feeding (IOF)	Age (AG)			Mean		*p*		
		1	7	14		SEM	IOF	AG	IOF × AG
Body weight (g)	Uninjected egg	53.49	161.24	452.47	222.40	0.60	0.4236	< 0.0001	0.7777
	Solution based (SB)	53.05	161.78	437.31	217.38				
	SB/chondroitin	49.96	149.38	456.81	218.71				
	SB/glucosamine	53.61	158.80	483.26	231.89				
	SB/chondroitin/glucosamine	51.17	147.74	441.97	213.63				
	Mean	52.25C	155.79B	454.36A					
Heart (%)	Uninjected egg	0.73Ba	0.76Aa	0.70Aa	0.72	0.03	0.0487	0.4242	0.0465
	Solution based (SB)	0.98Aa	0.80Ba	0.72Ba	0.83				
	SB/chondroitin	0.87ABa	0.79Aa	0.86Aa	0.84				
	SB/glucosamine	0.75Ba	0.80Aa	0.79Aa	0.78				
	SB/chondroitin/glucosamine	0.78Ba	0.92Aa	0.82Aa	0.84				
	Mean	0.82	0.81	0.78					
Liver (%)	Uninjected egg	1.99	3.71	4.13	3.28	0.07	0.4386	< 0.0001	0.7146
	Solution based (SB)	2.35	3.66	4.10	3.40				
	SB/chondroitin	2.10	3.93	4.03	3.35				
	SB/glucosamine	1.92	3.89	3.71	3.17				
	SB/chondroitin/glucosamine	2.26	4.01	4.38	3.55				
	Mean	2.12C	3.86B	4.07A					
Bursa (%)	Uninjected egg	0.10	0.16	0.92	0.39	0.01	0.3078	< 0.0001	0.1554
	Solution based (SB)	0.12	0.16	0.96	0.42				
	SB/chondroitin	0.10	0.16	0.89	0.38				
	SB/glucosamine	0.10	0.18	0.77	0.35				
	SB/chondroitin/glucosamine	0.14	0.15	1.00	0.45				
	Mean	0.11B	0.16B	0.91A					

Abbreviations: a–c, within columns differ (Tukey, *p* < 0.05); A–C, within rows differ (Tukey, *p* < 0.05); AG, age; IOF, in ovo feeding; SEM, standard error of the mean.

A significant interaction between in ovo feeding and age was observed for villus height in the jejunum (*p* < 0.0001). At 14 days of age, broilers subjected to the solution‐based treatment supplemented with chondroitin and glucosamine exhibited greater villus height compared with the other treatments (Table 3).

**TABLE 3:** Effects of in ovo feeding on jejunal morphometric parameters of broiler chickens at 1, 7, and 14 days of age.

	In ovo feeding (IOF)	Age (AG)			Mean		*p*		
		1	7	14		SEM	IOF	AG	IOF × AG
Villus height (μm)	Uninjected egg	176.40Ca	514.75Ba	665.75Ac	452.30	23.28	0.0240	< 0.0001	< 0.0001
	Solution based (SB)	256.41Ba	506.22Aa	539.28Ad	433.97				
	SB/chondroitin	210.88Ca	481.71Ba	759.16Ab	483.92				
	SB/glucosamine	213.95Ca	491.64Ba	687.75Ab	464.45				
	SB/chondroitin/glucosamine	234.48Ca	516.57Ba	853.15Aa	534.73				
	Mean	218.43	502.18	701.02					
Tip width (μm)	Uninjected egg	38.97	89.03	123.14	83.71	4.16	0.9210	< 0.0001	0.6367
	Solution based (SB)	43.54	115.20	111.95	90.23				
	SB/chondroitin	40.09	101.31	117.48	86.29				
	SB/glucosamine	42.07	93.40	128.37	87.95				
	SB/chondroitin/glucosamine	44.43	97.25	120.47	87.38				
	Mean	41.82C	99.24B	120.28A					
Villus base width (μm)	Uninjected egg	43.47	119.46	144.73	102.56b	5.69	0.0161	< 0.0001	0.2902
	Solution based (SB)	54.42	147.85	166.70	122.99a				
	SB/chondroitin	45.76	127.44	143.14	105.45b				
	SB/glucosamine	44.74	136.69	179.80	120.41a				
	SB/chondroitin/glucosamine	53.83	122.79	180.60	119.07a				
	Mean	48.44C	130.85B	162.99A					
Crypt depth (μm)	Uninjected egg	32.47	165.31	156.30	123.92a	6.35	0.0162	< 0.0001	0.1808
	Solution based (SB)	47.31	161.15	119.72	108.78b				
	SB/chondroitin	39.60	172.01	161.50	124.37a				
	SB/glucosamine	44.74	151.94	159.39	118.69a				
	SB/chondroitin/glucosamine	45.68	181.08	155.70	127.48a				
	Mean	42.79B	166.30A	151.58A					
Muscularis (μm)	Uninjected egg	72.56	98.16	138.47	103.06	3.79	0.2726	< 0.0001	0.3462
	Solution based (SB)	70.42	92.26	100.23	87.63				
	SB/chondroitin	64.76	92.02	127.50	94.76				
	SB/glucosamine	76.19	85.77	149.18	103.71				
	SB/chondroitin/glucosamine	74.50	109.09	135.07	106.22				
	Mean	71.69C	95.46B	130.09A					
Villus height/Crypt depth	Uninjected egg	6.04	3.24	4.54	4.61	0.13	0.9112	< 0.0001	0.4443
	Solution based (SB)	5.61	3.28	4.93	4.61				
	SB/chondroitin	5.56	2.93	5.00	4.50				
	SB/glucosamine	5.10	3.53	4.59	4.40				
	SB/chondroitin/glucosamine	5.35	3.04	5.26	4.49				
	Mean	5.53A	3.20C	4.84B					

Abbreviations: a–c, within columns differ (Tukey, *p* < 0.05); A–C, within rows differ (Tukey, *p* < 0.05); AG, age; IOF, in ovo feeding; SEM, standard error of the mean.

Age significantly affected jejunal villus tip width (*p* < 0.0001), villus base width (*p* < 0.0001), crypt depth (*p* < 0.0001), muscularis thickness (*p* < 0.0001), and the villus height‐to‐crypt depth ratio (*p* < 0.0001). All morphometric parameters increased with advancing age, except for the villus height‐to‐crypt depth ratio, which decreased over time (Table 3).

In ovo feeding influenced jejunal villus base width (*p* = 0.0161) and crypt depth (*p* = 0.0162). Lower villus base width values were observed in broilers from uninjected eggs compared with those receiving the solution‐based treatment. In contrast, lower crypt depth values were observed in broilers from the solution‐based treatment compared with uninjected eggs (Table 3).

In the ileum, a significant interaction between in ovo feeding and age was observed for crypt depth (*p* = 0.0437). Crypt depth increased with age across all treatments; however, at 7 days of age, lower values were observed in broilers from the solution‐based treatment, as well as those receiving glucosamine and the combination of chondroitin and glucosamine, compared with uninjected eggs (Table 4).

**TABLE 4:** Effects of in ovo feeding on ileal morphometric parameters of broiler chickens at 1, 7, and 14 days of age.

	In ovo feeding (IOF)	Age (AG)			Mean		*p*		
		1	7	14		SEM	IOF	AG	IOF × AG
Villus height (μm)	Uninjected egg	171.29	483.25	671.47	442.00	21.15	0.6044	< 0.0001	0.2199
	Solution based (SB)	188.71	491.77	561.77	414.08				
	SB/chondroitin	183.53	505.87	598.74	429.38				
	SB/glucosamine	156.53	452.10	670.14	426.26				
	SB/chondroitin/glucosamine	173.03	485.66	557.43	405.37				
	Mean	174.62C	483.73B	611.91A					
Tip width (μm)	Uninjected egg	43.18	95.98	120.27	85.92	4.06	0.0659	< 0.0001	0.2095
	Solution based (SB)	41.51	88.32	137.45	86.25				
	SB/chondroitin	46.15	86.89	126.69	86.58				
	SB/glucosamine	43.75	95.33	108.76	82.62				
	SB/chondroitin/glucosamine	49.87	107.13	141.89	99.63				
	Mean	44.89C	94.69B	126.65A					
Villus base width	Uninjected egg	52.71	154.80	162.09	123.20ab	5.75	0.0249	< 0.0001	0.0638
	Solution based (SB)	59.19	128.28	174.07	120.51ab				
	SB/chondroitin	57.76	116.41	166.20	113.45ab				
	SB/glucosamine	46.99	130.18	145.43	107.53b				
	SB/chondroitin/glucosamine	53.04	146.84	198.59	132.82a				
	Mean	53.94C	135.30B	169.27A					
Crypt depth (μm)	Uninjected egg	31.51Ba	166.66Aa	142.23Aa	113.47	5.75	0.5918	< 0.0001	0.0437
	Solution based (SB)	41.55Ca	131.25Bb	159.15Aa	110.65				
	SB/chondroitin	36.79Ba	141.47Aab	149.28Aa	109.15				
	SB/glucosamine	41.72Ca	115.98Bb	144.62Aa	100.77				
	SB/chondroitin/glucosamine	46.60Ba	125.13Ab	144.82Aa	105.52				
	Mean	39.61	136.10	148.02					
Muscularis (μm)	Uninjected egg	118.61	137.00	164.25	139.95	4.99	0.3199	< 0.0001	0.3286
	Solution based (SB)	94.43	108.49	204.51	136.81				
	SB/chondroitin	97.43	113.52	166.03	125.66				
	SB/glucosamine	94.63	105.13	164.26	121.34				
	SB/chondroitin/glucosamine	110.94	119.01	197.30	142.42				
	Mean	103.21B	116.63B	179.27A					
Villus height/crypt depth	Uninjected egg	5.75Aa	3.07Bc	5.08Aa	4.63	0.10	0.0252	0.0022	< 0.0001
	Solution based (SB)	4.87Ab	4.03Ab	3.67Bc	4.19				
	SB/chondroitin	5.16Aa	3.89Bb	4.38Ab	4.48				
	SB/glucosamine	4.10Ba	4.39Aa	5.36Aa	4.57				
	SB/chondroitin/glucosamine	3.85Ba	4.10Ab	4.09Ab	4.01				
	Mean	4.74	3.90	4.49					

Abbreviations: a–c, within columns differ (Tukey, *p* < 0.05); A–C, within rows differ (Tukey, *p* < 0.05); AG, age; IOF, in ovo feeding; SEM, standard error of the mean.

A significant interaction between in ovo feeding and age was also observed for the villus height‐to‐crypt depth ratio in the ileum (*p* < 0.0001). At 1 day of age, this ratio was higher in broilers from uninjected eggs than in those receiving the solution‐based treatment supplemented with chondroitin. At 7 days of age, higher values were observed in broilers from the solution‐based treatment, glucosamine, and the combination treatment, except for the chondroitin treatment. At 14 days of age, higher values were mainly observed in broilers from the glucosamine treatment and uninjected eggs (Table 4).

Ileal villus height, villus tip width, villus base width, and muscularis thickness were significantly affected by age (*p* < 0.0001), with values increasing as birds aged. In ovo feeding also affected ileal villus base width (*p* = 0.0249), with lower values observed in broilers from uninjected eggs and those receiving glucosamine compared with the combination treatment (Table 4).

A significant interaction between in ovo feeding and age was observed for the relative abundance of the cadherin‐1 (CDH1) gene (*p* = 0.0292). At 1 day of age, higher relative abundance of the CDH1 gene was observed in broilers from the solution‐based treatment and those receiving the combination of chondroitin and glucosamine (Table 5). At 7 days of age, higher values were observed in broilers from the combination treatment. At 14 days of age, higher relative abundance of the CDH1 gene was observed in broilers from the glucosamine treatment and the combination treatment. A reduction in relative abundance of the CDH1 gene with advancing age was observed only in broilers from the solution‐based treatment (Table 5).

**TABLE 5:** Effects of in ovo feeding on the relative abundance of the cadherin‐1 (CDH1) gene in broiler chickens at 1, 7, and 14 days of age.

	In ovo feeding (IOF)	Age (AG)			Mean		*p*		
		1	7	14		SEM	IOF	AG	IOF × AG
Relative abundance of the cadherin‐1 (CDH1) gene	Uninjected egg	1.00Bb	17.07Aab	14.91Aab	10.99	3.98	0.1381	0.2775	0.0292
	Solution based (SB)	27.80Aa	10.60ABb	2.37Bb	13.59				
	SB/chondroitin	6.31Aa	11.63ABb	1.45Bb	6.46				
	SB/glucosamine	3.12Bb	9.61ABb	24.80Aa	12.51				
	SB/chondroitin/glucosamine	23.12Aa	26.34Aa	20.23Aa	23.23				
	Mean	12.27	15.05	12.75					

Abbreviations: a–c, within columns differ (Tukey, *p* < 0.05); A–C, within rows differ (Tukey, *p* < 0.05); AG, age; IOF, in ovo feeding; SEM, standard error of the mean.

## 4 Discussion

This study evaluated the effects of in ovo administration of glycosaminoglycans, ascorbic acid, pyridoxine, amphotericin, and gentamicin on incubation characteristics, intestinal morphometry, organ development, and cadherin‐1 (CDH1) gene relative abundance in broilers.

The absence of significant effects on hatching rate, embryo mortality, and total mortality indicates that in ovo feeding performed at 17.5 days of incubation did not compromise embryonic viability. According to Uni et al. (2005), excessive osmolarity of injected solutions may disrupt embryonic osmotic balance and impair hatchability, with a maximum recommended osmolarity of 800 mOsm for in ovo administration. The lack of negative effects observed in the present study suggests that the composition and volume of the injected solutions were within a physiologically tolerable range.

Similarly, Santos et al. (2018) reported that in ovo injection of glycosaminoglycans did not affect hatching or embryonic development, supporting the safety of these compounds during incubation. In contrast, Sgavioli et al. (2015) observed reduced hatchability when higher concentrations of vitamin C were injected before incubation, emphasizing that the effects of in ovo feeding depend on nutrient concentration, embryonic developmental stage, and injection site.

As expected, advancing age significantly increased body weight, relative organ weights, villus height, villus width, muscularis thickness, and crypt depth, while reducing the villus height‐to‐crypt depth ratio. These responses are consistent with the normal physiological growth and maturation of the gastrointestinal tract in broilers, as previously described by Silva et al. (2003). Intestinal maturation during early life is critical for nutrient digestion and absorption and plays a central role in determining subsequent growth performance.

Cardiovascular development at hatch is a key determinant of posthatch adaptation, as a relatively larger heart may improve circulatory efficiency and reduce susceptibility to metabolic disorders such as ascites during later growth stages (Molenaar et al. 2011). In the present study, broilers from the solution‐based treatment exhibited a higher relative heart weight at 1 day of age.

This transient response may reflect enhanced metabolic support and tissue growth during late embryogenesis rather than pathological cardiac overload (Marsico et al. 2023). Importantly, this effect was not sustained at later ages, suggesting a temporary developmental modulation rather than long‐term alteration of cardiac structure. Similar to the findings of Sgavioli et al. (2013), who reported no persistent effects of in ovo ascorbic acid injection on heart weight, the present results indicate that further studies are necessary to clarify the functional implications of early changes in cardiac development.

This study is the first to evaluate the effects of glycosaminoglycans administered via in ovo feeding on intestinal morphometry and CDH1 gene relative abundance in broilers. Although the responses observed in jejunal and ileal morphometric parameters were not entirely consistent across ages, some positive effects were observed in birds receiving the solution‐based treatment, particularly when supplemented with chondroitin and glucosamine. These birds exhibited greater villus height and, at specific ages, higher villus height‐to‐crypt depth ratios, suggesting potential improvements in intestinal structural development during early posthatch life.

Glucosamine and chondroitin are well recognized for their anti‐inflammatory properties and are classified as slow‐acting symptomatic agents (Brandt, 2002). In addition to their systemic effects, these compounds may influence intestinal health by modulating the gut microbiota, reducing the abundance of pathogenic microorganisms, and supporting epithelial homeostasis (Shang et al. 2016; Liu et al. 2017). A healthier intestinal microbiota contributes to improved intestinal integrity, which is closely associated with favorable histomorphometric characteristics and enhanced nutrient utilization (Boleli et al. 2017).

The beneficial effects of glucosamine sulfate on epithelial defense mechanisms and intestinal morphology have been previously demonstrated in humans (Salvatore et al. 2000) and are consistent with the intestinal responses observed in the present study. Early‐life development of the intestinal mucosa in broilers is particularly important, as processes such as enterocyte proliferation, mucosal differentiation, and crypt‐villus organization directly influence feed efficiency and growth throughout the production cycle (Boleli et al. 2017).

Consistent with previous findings, Martins et al. (2020) demonstrated that dietary supplementation with glycosaminoglycans improves intestinal histomorphometry, nutrient digestibility, and performance in broilers. These effects are especially relevant in the jejunum and ileum, which represent the primary sites for nutrient digestion and mineral absorption (Mutucumarana et al. 2014).

In the present study, in ovo feeding influenced the relative abundance of the CDH1 gene, particularly in treatments supplemented with glycosaminoglycans. Cadherin‐1 plays a fundamental role in cell–cell adhesion and tissue organization and is expressed in osteogenic precursors (Kawaguchi et al. 2001), where it contributes to osteoblast function (Wan et al. 2011), osteoclast attachment to the extracellular matrix (Ilvesaro et al. 1998), and precursor cell fusion (Mbalaviele et al. 1995). The modulation of CDH1 gene relative abundance observed herein suggests a potential involvement of glycosaminoglycans in cellular adhesion and tissue organization processes during early development. However, it is important to note that CDH1 alone does not fully represent intestinal barrier function, which also depends on tight junction proteins, apoptosis‐related pathways, and other regulatory mechanisms not evaluated in this study.

The variability in responses to in ovo feeding treatments highlights the complexity of glycosaminoglycan actions on broiler physiology (Das et al. 2021; Kpodo and Proszkowiec‐Weglarz 2023). Although some positive effects were observed on intestinal morphometry and CDH1 gene relative abundance, the underlying biological mechanisms remain incompletely understood and warrant further investigation. Future studies integrating functional cardiac assessments, additional intestinal barrier markers, and microbiota profiling may provide deeper insight into the pathways involved.

Overall, these findings reinforce the potential of glycosaminoglycans as bioactive compounds in broiler production and support the use of in ovo feeding as a strategy to modulate early intestinal development. Nonetheless, additional research is needed to optimize nutrient combinations, administration protocols, and to clarify long‐term impacts on broiler performance and health.

## 5 Conclusions

In ovo feeding with glycosaminoglycans, ascorbic acid, pyridoxine, amphotericin, and gentamicin modulated early intestinal morphometry and transiently increased relative heart weight in broilers, without impairing hatching rate or embryo survival. These findings indicate that in ovo feeding with glycosaminoglycan‐based solutions represents a safe strategy to influence early posthatch development in broilers. However, further studies are required to clarify the underlying mechanisms and long‐term functional implications.

## Author Contributions

**Elaine Talita Santos:** conceptualization, investigation, methodology. **Sarah Sgavioli:** writing – original draft, writing – review and editing. **Ramon Diniz Malheiros:** formal analysis, data curation. **Silvana Martinez Baraldi‐Artoni:** conceptualization, methodology. **Melody Martins Cavalcante Pereira, Rodrigo Garófallo Garcia, Jean Kaique Valentim:** writing – review and editing. **Peter Ferket:** supervision.

## Conflicts of Interest

The authors declare no conflicts of interest.
